# The relation between cesarean birth and child cognitive development

**DOI:** 10.1038/s41598-017-10831-y

**Published:** 2017-09-13

**Authors:** Cain Polidano, Anna Zhu, Joel C. Bornstein

**Affiliations:** 1 Melbourne Institute of Applied Economic and Social Research, Level 5, Faculty of Business and Economics Building, University of Melbourne, Carlton, 3010 Australia; 2 Department of Physiology, Level 6, North Wing, Medical Building, University of Melbourne, Carlton, 3010 Australia

## Abstract

This is the first detailed study of the relation between cesarean birth and child cognitive development. We measure differences in child cognitive performance at 4 to 9 years of age between cesarean-born and vaginally-born children (n = 3,666) participating in the Longitudinal Study of Australian Children (LSAC). LSAC is a nationally representative birth cohort surveyed biennially. Using multivariate regression, we control for a large range of confounders related to perinatal risk factors and the socio-economic advantage associated with cesarean-born children. Across several measures, we find that cesarean-born children perform significantly below vaginally-born children, by up to a tenth of a standard deviation in national numeracy test scores at age 8–9. Estimates from a low-risk sub-sample and lower-bound analysis suggest that the relation is not spuriously related to unobserved confounding. Lower rates of breastfeeding and adverse child and maternal health outcomes that are associated with cesarean birth are found to explain less than a third of the cognitive gap, which points to the importance of other mechanisms such as disturbed gut microbiota. The findings underline the need for a precautionary approach in responding to requests for a planned cesarean when there are no apparent elevated risks from vaginal birth.

## Introduction

Cesarean sections can save lives, but rates well above the World Health Organization’s recommended 15% ceiling in most developed countries suggest that many procedures are unnecessary^1^. In the OECD, the rate of caesarean section has increased from 14.4% of deliveries in 1990 to 25.8% in 2009^2^, growth that cannot be explained by increases in obstetric risk factors, including those associated with delayed and multiple infant pregnancy and maternal obesity^3–6^. Instead, this growth is believed to be related to increased maternal request and changes in clinical practice^7^. A concern is that many procedures are made without considering possible long-run implications, such as reduced fertility^8^. In this study, we concentrate on examining possible long-term implications for child cognitive development.

Cesarean birth may be directly and indirectly associated with negative child cognitive outcomes. The indirect association may occur through established links between cesarean birth and adverse child health outcomes, including asthma, type I diabetes, allergies^9–11^ and obesity^12^ that are also associated with impaired functioning and lower academic performance^13, 14^. The surgical nature of cesarean procedures also poses postnatal maternal health risks^15^, with potential knock-on effects for the child’s development through altered mother-child interactions^16^ and lower rates of breastfeeding^17–19^.

The direct association may occur through alterations to the infant’s gut microbiota. Unlike vaginally-born children whose gut is seeded by passing through the birth canal, the gut of cesarean-born children is seeded through contact with the mother’s skin and hospital surfaces. The result is long-term compositional differences in gut microbiota by mode of birth^20–24^, with differences observed up until age seven^24^. Recently uncovered chemical signaling from the gut microbiota to the central nervous system, affecting memory, motivation, mood and stress reactivity, raises questions about the long-term cognitive effects of disturbed microbiota composition at a sensitive time in brain development^25, 26^. Although causal impacts on child development are yet to be proven, altered signaling from disturbed gut microbiota is thought to be a possible driver of higher rates of cognitive disorders, especially autism spectrum disorder (ASD) and attention deficit hyperactivity disorder (ADHD), among cesarean-born children (see Curran *et al*.^27^ for a review). In animal studies, there is mounting (and replicated) evidence of long-term cognitive impacts from early microbiota disturbance. These studies show that rodents lacking all gut bacteria from birth (germ-free animals) have memory deficits^28^ and behavioral abnormalities^29, 30^ when compared to animals with normal gut microbiota. Interestingly, these cognitive deficits in rodents can be corrected through the colonization of the intestine via a fecal transplant from the control group, but only if it occurs early in life. The implication is that there is a critical window to correct any disruption to the gut microbiota, after which cognitive impacts are permanent^25^.

In this study, we estimate the relation between cesarean birth and child cognitive outcomes using data from the Longitudinal Study of Australian Children (LSAC), a nationally representative birth cohort surveyed biennially, and multivariate regression techniques. To the best of our knowledge, only Bentley *et al*.^31^ has examined the relation between cesarean birth and cognitive outcomes. Using Australian hospital records linked to teacher-based assessments in the first year of school, they found a significant negative association across several developmental domains. We build on this study and that of previous health studies in several ways. First, we build on the work of Bentley *et al*.^31^ by using mediation analysis to test to what extent any relation is associated with lower rates of breastfeeding and adverse child and maternal health outcomes. This is important because it helps to identify the importance of direct effects, such as those related to disturbed gut microbiota and helps identify how widespread any effects may be within the population. Second, LSAC contains rich longitudinal cognitive test information that enables us to gain an insight into longer-term outcomes that are closely related to early academic achievement, an important predictor of lifelong earnings and health. These include academic test results that measure: school readiness at 4–5, vocabulary and comprehension at 4–5, 6–7 and 8–9 years, problem solving at 6–7 and 8–9 years and national achievement in numeracy and literacy at 8–9 years. Third, we more rigorously test the sensitivity of any relation to bias from confounding variables not observed in the data or ‘selection on unobserved covariates’. We do this using two methods — re-estimating on a sub-sample where selection on unobserved covariates is likely to be less of an issue (privately insured births without any observed perinatal risk factors) and by estimating lower-bound estimates under conservative assumptions about the magnitude of selection on unobserved covariates using the Oster^32^ technique. This helps to provide some guidance on whether estimated relation is plausibly causal.

## Methods

### Data

LSAC is an internationally recognized and widely-used longitudinal cohort survey of child health and development^33, 34^. There are multiple cohorts of LSAC that are designed for examining specific development periods. In this study, we use the LSAC ‘B cohort’, which has an initial sample of around 5,100 infants born between March 2003 to February 2004, drawn randomly from all registered national births at the time. The initial survey, conducted between 6 months and a year after birth, contains rich perinatal information about the mother and study child along with detailed information on the parents’ background and current life circumstances. From the initial survey, children and their primary care giver are surveyed biennially. At the time of analysis, data were only available up until wave 5 (2014) of the survey when the children are 8–9.

Of the initial 5,100 LSAC participants, we omit around 1,300 from the sample because of missing information by wave 5, due mainly to survey attrition. To examine the potential impact of attrition, for two measures that are observed in wave 3 (Peabody Picture Vocabulary and Who Am I?), we compare relations estimated using the full sample available at wave 3 and relations estimated at wave 3 on a restricted sample that remain in the survey until wave 5 (n = 3,666). Using a two-sample t-test, we find no evidence that results estimated results at wave 3 are different for the full and restricted samples (${\chi }^{2}$ = 0.54, p-value = 0.46 and ${\chi }^{2}$ = 1.64, p-value = 0.20 respectively). The implication is that non-random attrition does not appear to be seriously biasing our results.

Whether participants in LSAC are cesarean born is identified in the initial survey by the primary care giver’s response to the question, “was…the type of birth/ delivery method a cesarean?” In our sample, approximately 30% of primary care givers report that their children were cesarean-born (between March 2003 and February 2004). To the extent that there is a stigma associated with elective procedures, a potential concern is that the rate of cesarean birth may be under-reported in LSAC (social desirability bias). However, this is not borne out in national statistics from hospital records that show comparable rates of cesarean birth — 28% and 30% for 2003 and 2004 respectively^35, 36^. Compared to other OECD countries, Australia’s rate is similar to that of the United States and Germany, but lower than in Italy (38%) and Brazil (54%) and higher than Finland, Norway and Sweden (around 17%)^37^.

### Data availability

No data were specifically collected for this project. The data used was previously collected by the Australian Institute of Family Studies (AIFS) and was conducted in full accordance with all relevant guidelines and regulations as spelt out in the AIFS Ethics Committee approval. This includes obtaining consent for survey participation and use of anonymized information for research purposes, as approved by AIFS. AIFS provided anonymized data for this project under an individual license agreement. In the analysis and in preparing the manuscript, we have adhered to all requirements under the license agreement. Data can be made available upon request to the corresponding author, subject to approval from AIFS.

### Outcomes

#### Measures of cognitive development

There are two types of cognitive development measures in LSAC. The first are scores from three interviewer-administered tests conducted between ages 4 and 9 that are part of the LSAC survey and the second are scores from a national standardized test in numeracy and literacy at age 8–9 that are matched to LSAC participants. All cognitive measures are age-normalized and standardized with respect to the weighted sample mean and standard deviation (descriptive statistics in Table 1 are age normalized, but unstandardized).

**Table 1:** Sample mean values for main control variables in LSAC B cohort.

	Mode of birth				p-value of diff.^a^
	Cesarean	*s.e.*	Vaginal	*s.e.*	
	(n=1,080)		(n=2,586)		
**Dependent variables** ^**b**^					
*Survey-based measures*					
School preparedness (WAI), 4–5	65.3	5.9	65.8	6.0	[0.112]
Vocabulary (PPVT), 4–5	65.4	8.8	65.3	8.4	[0.784]
Vocabulary (PPVT), 6–7	74.8	5.1	74.5	5.0	[0.134]
Vocabulary (PPVT), 8–9	79.5	4.6	79.3	4.9	[0.143]
Problem solving (MR), 6–7	10.8	3.1	10.8	3.0	[0.601]
Problem solving (MR), 8–9	10.5	2.9	10.6	3.0	[0.673]
*Grade 3 NAPLAN*					
Numeracy	409.2	72.9	412.0	74.8	[0.359]
Reading	436.3	90.5	439.4	90.0	[0.395]
Writing	423.2	62.0	425.7	59.5	[0.313]
Spelling	419.4	78.4	420.9	77.9	[0.641]
Grammar	438.8	96.9	441.6	91.9	[0.481]
**Controls** ^**c**^					
*Family characteristics (socio-economic status (SES)) in year of birth*					
Maternal age at birth	31.752	4.810	30.438	5.159	[0.000]
Maternal age at birth squared	1031	307	953	313	[0.000]
Three or more older siblings	0.041	0.198	0.073	0.261	[0.000]
Female child	0.457	0.498	0.500	0.500	[0.019]
Either parent was born in a disadvantaged country^d^	0.046	0.210	0.071	0.257	[0.002]
Mother is single	0.051	0.220	0.068	0.252	[0.040]
Mother is not legally married to partner	0.804	0.397	0.769	0.421	[0.018]
Mother was employed	0.532	0.499	0.532	0.499	[0.969]
Mother’s highest qualification					
High school diploma or below^e^	0.256	0.436	0.292	0.455	[0.022]
Vocational education qualification^f^	0.261	0.439	0.263	0.441	[0.889]
College (bachelor) degree or above	0.483	0.500	0.444	0.497	[0.031]
Private health insurance	0.591	0.492	0.488	0.500	[0.000]
*Perinatal risk factors (PN)*					
Low birthweight (<2.5 kg)	0.066	0.248	0.034	0.181	[0.000]
IVF treatment used	0.085	0.279	0.052	0.222	[0.000]
Multiple births	0.064	0.245	0.017	0.128	[0.000]
Head circumference of child (Z-score)^g^	−0.262	1.119	−0.326	0.871	[0.090]
Length of baby when born (Z-score)^g^	0.054	1.382	0.377	0.988	[0.000]
Blood pressure medication during pregnancy	0.030	0.170	0.016	0.126	[0.020]
Diabetes medication during pregnancy	0.021	0.144	0.006	0.076	[0.001]
Antibiotic medication during pregnancy	0.108	0.311	0.098	0.297	[0.345]
Weeks of gestation	38.563	2.084	39.462	1.695	[0.000]
*State of residence*					
New South Wales	0.279	0.449	0.320	0.466	[0.013]
Victoria and Australian Capital Territory	0.281	0.450	0.272	0.445	[0.553]
Western Australia	0.117	0.321	0.097	0.297	[0.091]
Queensland	0.212	0.409	0.200	0.400	[0.411]
South Australia	0.076	0.265	0.071	0.256	[0.588]
Northern Territory	0.009	0.096	0.018	0.132	[0.029]
Tasmania	0.026	0.159	0.022	0.148	[0.536]
Outside metropolitan areas	0.348	0.477	0.375	0.484	[0.121]

^a^The p-values are associated with a t-test for differences in mean values between cesarean and vaginally born children. ^b^The descriptive results above are age-normalised, but unstandardized to give the reader a fuller picture of the data. In the regression analysis, all cognitive performance measures are age-normalised and standardised with respect to the weighted sample mean and standard deviation. ^c^All control variables are taken from wave 1 of the data (2004) when children are 0–1 year of age. ^d^Disadvantaged countries are those identified by the Australian Bureau of Statistics Standard Australian Classification of Counties (2011), ABS cat. no. 1269.0. ^e^High school diploma includes vocational equivalent — International Standard Classification of Education (ISCED) 1997 level 3 C. ^f^Vocational education qualification is ISCED 1997 level 4B and 5 A. ^g^Z-scores are based on Centre for Disease Control and Prevention (CDC) growth charts and are age and gender-adjusted.

The interviewer-administered cognitive tests from LSAC B are the Peabody Picture Vocabulary Test (PPVT)^38^; Who Am I? (WAI)^39^ and the Matrix Reasoning test (MR)^40^. The PPVT is an age appropriate vocabulary test designed to measure a child’s knowledge of the meaning of spoken words and their comprehension and ability to respond. The test was carried out in survey when the children were aged 4–5, 6–7 and 8–9. WAI is an assessment of the child’s readiness for school and measures the child’s ability to perform a range of tasks, such as reading, writing, copying, and symbol recognition. WAI was only carried out when the children were 4–5. Finally, MR is a test of problem solving ability, based on the Wechsler Intelligence Scale for Children. The test is age appropriate and was conducted when the children were aged 6–7 and 8–9.

The national standardized tests are from the National Assessment Program for Literacy and Numeracy (NAPLAN). NAPLAN is conducted in all Australian schools to measure performance in numeracy and literacy at grades 3, 5, 7 and 9, corresponding to ages 8–9, 10–11, 12–13 and 14–15. In the case of literacy, performance is measured over the domains of reading, writing, grammar and spelling. Each student’s performance in NAPLAN, including their national ranking compared to similar-age students, is made available to schools and students to monitor performance. At the time of analysis, only NAPLAN data for grade 3 was available for the LSAC B cohort. For students who were not in grade 3 at the time of testing, usually because they commenced school at a later age than the rest of the cohort, we imputed their values. The imputation process involved two steps. The first step involved using the sample with observed grade 3 NAPLAN scores to estimate regression model relations between grade 3 NAPLAN scores and personal information (such as other interviewer-administered cognitive tests). In the second step, we use results from the first step to generate predicted NAPLAN values for those with missing scores, that is, we apply the estimated regression model relations to the personal characteristics of those with missing NAPLAN scores^41^. Imputing these values instead of omitting them makes little difference to our analysis (see Table S1 of the online supporting material).

#### Mediators

In this study we measure the extent to which any relation between cesarean birth and cognitive development is mediated by lower rates of breastfeeding and adverse child and maternal health outcomes. We selected these variables because they have been previously associated with cesarean birth and are available in the data^9–19^. Adverse child health outcomes include measures of obesity and care giver reported diagnosis of asthma, ADD or ASD. While measures of asthma and obesity are available at the time of cognitive testing, measures for ADD and ASD are only available at ages 6–7 and 8–9 years. Because only around two-thirds of care givers answer questions related to ADD and ASD at age 6–7, we use information at age 8–9 years when the response rate is much higher. Obesity is measured by whether the child’s Body Mass Index (BMI) is above the upper-limit of normal range of 19.3 for those 4–7 years and 23 for 8–9 years. Breastfeeding and maternal health measures are from the initial sample (6–12 months after birth). Breastfeeding is a binary measure of whether the child was breastfed at three months or not. Two self-reported postnatal maternal health measures were used: whether any depressive symptoms were experienced in the last 4 weeks (score of below 3 on a 6-point Kessler scale) and whether general health was reported as fair to poor (4 or 5 on a 5-point scale, where 1 is excellent health).

### Controls

The analysis includes over 20 confounders grouped into two main categories (Table 1): those related to perinatal risk factors and those related to the socio-economic advantage associated with cesarean-born children in Australia. Perinatal risk factors include the taking of medication during pregnancy for blood pressure or diabetes (proxies for pre-eclampsia and gestational diabetes respectively), the taking of antibiotic medication (a proxy for bacterial infection, which may also affect the development of the infant’s gut microbiome); a dummy variable for low birth weight (coded 1 if less than 2.5 kg; 0 otherwise); weeks of gestation; maternal age at birth; dummy variable for multiple infant pregnancy; length and head circumference of baby (z-scores); dummy variable for whether the baby was conceived using IVF treatment and a gender dummy. We include taking antibiotic medication as a control because it has been associated with changes to the infant’s gut microbiome^42^ and possibly the risk of cesarean birth, which means failure to control for it will lead to bias due to unobserved confounding. However, we stress that our results are not sensitive to the inclusion of this control, or other child and maternal health risks in the data (refer to results for the ‘low-risk privately insured’ group in Table 2). Length and head circumference z-scores are based on Centre for Disease Control and Prevention (CDC) growth charts and are age and gender-adjusted. We also estimated a model with an alternative treatment of low birth weight (below 1.5 kg) and a model on a sub-sample of births with gestational ages of 37–40 weeks using binary dummy variable controls for each gestational week (excluding the reference category of 40 weeks). Results for these alternative models are much the same as those reported in Table 2 (available upon request from the corresponding author).

**Table 2:** OLS regression estimates and Oster lower-bound estimates of the relations between cesarean birth and child cognitive (standard deviations).

Child cognitive measures	OLS regression				Oster^b^
	All births		Low-risk privately insured births^a^		Coefficient lower-bound
	Coefficient	*s.e*.	Coefficient	*s.e*.	
*NAPLAN at 8–9*					
Reading	−0.076**	(0.033)	−0.051	(0.059)	−0.067
Writing	−0.060**	(0.031)	−0.131**	(0.053)	−0.055
Spelling	−0.043	(0.033)	−0.132**	(0.060)	−0.033
Grammar	−0.055*	(0.033)	−0.097	(0.061)	−0.042
Numeracy	−0.095***	(0.034)	−0.135**	(0.060)	−0.09
*LSAC survey administered measures*					
School readiness 4–5	−0.051	(0.035)	−0.127**	(0.061)	−0.014
Vocabulary 4–5	−0.087**	(0.035)	−0.127**	(0.057)	−0.092
Vocabulary 6–7	−0.05	(0.037)	−0.097	(0.064)	−0.056
Vocabulary 8–9	−0.047	(0.037)	−0.058	(0.067)	−0.054
Problem solving 6–7	−0.036	(0.117)	−0.052	(0.071)	−0.000
Problem solving 8–9	−0.069*	(0.039)	−0.077	(0.069)	−0.068

***p-value < 0.01; **p-value < 0.05; *p-value < 0.1. *SE*, standard error. Results are estimated with adjustments for confounders related to family socio-economic and perinatal risk factors. ^a^Low-risk privately insured births are those that are not low birth weight (at least 2.5 kg), full-term (38–40 weeks), singleton, conceived without IVF and without medication taken for blood pressure or diabetes during pregnancy and whose parents were privately insured in the year of birth. ^b^The Oster coefficient lower-bound is the estimated coefficient when adjustment in the coefficient due to selection on unobserved covariates is equal to adjustment due to selection on observable covariates.

Postnatal interventions such as the use of a ventilator and the use of intensive care were not included as controls because they may be considered an outcome of delivery mode. We also refrain from including prenatal risk factors available in the data that have a high rate of missing observations. These include an identifier for whether the mother is a regular smoker, maternal average consumption of more than two standard drinks a day and maternal body mass index outside of normal range (18.5 to 25). Excluding postnatal interventions and prenatal risk factors with high rates of missing observations makes little difference to the results (see Table S2 of the online supporting material for model results with these factors included as additional controls).

In general, descriptive statistics of the control variables presented in Table 1 show that cesarean birth is associated with higher perinatal risk factors and a socio-economic advantage, especially higher maternal education, fewer older siblings, lower rates of un-partnered birth and higher rates of private health insurance. Despite Australia’s universally available free public health system, many high income earners in Australia choose to hold private health insurance for two reasons. First, it provides a greater coverage of medical treatments, especially for allied health services; and second, it enables them to circumvent the payment of an income-contingent levy that to help meet the cost of the public health system. Important in the context of this study, maternal requested cesarean birth (without any medical risk factors) is not covered by the public health system. While mothers without private health insurance can still elect for cesarean birth in a public hospital, this is uncommon because they would incur all medical costs. It is much more common for elective cesareans to occur in private hospitals under the cover of private health insurance. This explains the 11 percentage point higher rate of private health insurance among cesarean born children than among vaginally born children.

### Statistical method

In our main analysis, we use OLS multivariate regression models for each of the cognitive measures to estimate the relation between cesarean birth and cognitive development. These models are of the following form:

1 $${Y}_{i}={\gamma }_{0}+{\gamma }_{1}C{S}_{i}+SE{S}_{i}{\gamma }_{2}+P{N}_{i}{\gamma }_{3}+{\upsilon }_{i}$$

where $C{S}_{i}$ equals one if child *i* was cesarean-born, 0 otherwise, $SE{S}_{i}$ and $P{N}_{i}$ are vectors of family socio-economic status and perinatal characteristics respectively (from Table 1) and ${\upsilon }_{i}\,$is an error term.

To try and explain the importance of possible channels, we measure the extent to which ${\gamma }_{1}$is mediated by lower rates of breastfeeding and adverse child and maternal health outcomes in the data. Mediating effects are estimated using the product of the coefficients method^43^.

Identification of the main parameter of interest, ${\gamma }_{1}$ in equation (1) and the mediating effects is complicated by potential unobserved confounding, which means that ${\gamma }_{1}$ may be biased by correlation between $C{S}_{i}$ and ${\upsilon }_{i}$. The main potential sources of unobserved confounding may be missing controls for perinatal risk factors (such as oxygen deprivation during birth) and socio-economic advantage (such as greater household income) associated with cesarean-born children. The presence of the former will lead to an over-estimate of any true negative relation between cesarean birth and cognitive development; whereas the presence of the latter will lead to an under-estimate of any negative relation.

Without the possibility for randomization, a common approach for dealing with this form of bias is instrumental variables. This method relies on the presence of factors in the data that affect cognitive development only through altering the chances of cesarean birth. We are unaware of any strong candidates in our data and we instead concentrate on testing the sensitivity of our results to bias from the presence of selection on unobserved covariates. These are discussed in detail below.

#### Sensitivity to unobserved perinatal confounders

We use two approaches to test the sensitivity of our OLS estimates to bias from unobserved perinatal covariates. First, we re-estimate OLS relations (using equation (1)) on a sub-sample of 2,140 births that are free of any observed health risk that may lead to cesarean birth and that are privately insured; termed, ‘low-risk privately insured’ group. The idea is that by restricting the sample to those that are more similar on observed covariates, we are reducing bias by also restricting differences in unobserved covariates. The ‘Low-risk privately insured’ sub-sample are those that are not low birth weight (above 2,500 grams), full-term (38–40 week gestation), singleton, conceived without IVF, whose mother took no blood pressure or diabetes medication during pregnancy and whose parents were privately insured in the year of birth. These models include all other covariates included in the full sample. We omitted those *without* private health insurance because they are more likely than those *with* private insurance to have a cesarean performed for medical reasons.

In the second approach, we use the Oster^32^ method, which like approaches proposed by Rosenbaum^44^ and Altonji^45^, bounds the relation under assumptions about selection on unobserved covariates (see Nghiema, Nguyen, Khanam and Connelly^46^ and Hanushek, Schwerdt, Woessmann and Zhang^47^ for recent applications of the Oster method). Under the Oster method, we estimate the lower bound of the OLS relation between cesarean birth and measures of child cognitive development using the following:

2 $${{\rm{\gamma }}}_{1,{\rm{lower}}}={\rm{f}}({{\rm{\gamma }}}_{1}-{{\rm{\gamma }}}_{1{\rm{u}}},\,{{\rm{R}}}^{2}-{{\rm{R}}}_{{\rm{u}}}^{2};\,{{\rm{R}}}_{{\rm{\max }}}^{2}-{{\rm{R}}}^{2},\,{\rm{\delta }}).$$

Variables ${{\rm{\gamma }}}_{1{\rm{u}}}$ and ${{\rm{R}}}_{{\rm{u}}}^{2}$ are results from the ‘uncontrolled’ model, which is equation (1) estimated without perinatal and socio-economic controls; ${{\rm{R}}}_{{\rm{\max }}}^{2}$ is theoretical maximum R-squared, or the maximum proportion of variation in the outcome variable that can be explained by the model; and ${\rm{\delta }}$ is the coefficient of proportionality, or the ratio of selection on unobserved covariates to selection on observed covariates:

3 $$\delta =\frac{cov({W}_{U},CS)}{cov({W}_{O},CS)}.\frac{var({W}_{O})}{var({W}_{U})},$$

where ${{\rm{W}}}_{{\rm{O}}},\,{{\rm{W}}}_{{\rm{U}}}$ are vectors of linear combinations of observed and unobserved covariates weighted by their true coefficients (or $\,{W}_{O}=\sum _{j=1}^{{J}_{O}}{\omega }_{j}^{O}{\gamma }_{j}^{O}$ and ${W}_{U}=\sum _{j=1}^{{J}_{U}}{\omega }_{j}^{U}{\gamma }_{j}^{U}$). Following Oster conventions, we set ${\rm{\delta }}=1,$ or estimate ${{\rm{\gamma }}}_{1,{\rm{lower}}}$ under the conservative assumption that selection on unobserved covariates is equal to selection on observed covariates and set ${{\rm{R}}}_{{\rm{\max }}}^{2}=1.3{{\rm{R}}}^{2}$. Setting ${{\rm{R}}}_{{\rm{\max }}}^{2}=1.3{{\rm{R}}}^{2}$ contrasts with the closely related Altonji^45^ method that assigns ${{\rm{R}}}_{{\rm{\max }}}^{2}=1$. Oster^32^ argues that assuming a value of 1 is unreasonable in the presence of measurement error. Under equation (2), the higher the proportion of variation in the outcome variable explained by the model (${{\rm{R}}}^{2})$, the smaller the discrepancy in the estimates of ${\gamma }_{1}\,$and ${{\rm{\gamma }}}_{1,{\rm{lower}}}$.

For any estimated negative relation between cesarean birth and cognitive development, the larger the discrepancy between estimates of ${{\rm{\gamma }}}_{1,{\rm{lower}}}$ and ${\gamma }_{1}$, the more unreliable ${\gamma }_{1}$ as an estimate of the true relation.

## Results

The estimated OLS relations between cesarean birth and child cognitive outcomes (equation (1)) for all births in our sample (n = 3,666) are reported in Table 2. The estimated coefficients for the control variables in this model can be found in Tables S3a and S3b of online supporting material. Generally speaking, the signs of these control variable coefficients are as expected, with the largest effects associated with maternal socio-economic factors. Specifically, we find that child cognitive outcomes are positively associated with mothers that are college educated (3-year bachelor degree), who give birth at an older age, who are partnered, who have private health insurance, are employed and have fewer previous births. The estimated coefficients of perinatal risk factors are generally insignificant or smaller and of mixed sign. Specifically, we find that taking blood pressure medication during pregnancy is associated with significant lower cognitive outcomes, while greater head circumference and body length is associated with positive cognitive outcomes. Consistent with previous studies, we find evidence, albeit only for select child cognitive outcomes, of a positive association with gestational age^31, 48^ and a negative association with low body weight (less than 2.5 kg)^49^. For the former, the association is only significant for school readiness at 4–5 and for the latter; the association is only significant for school readiness at 4–5, vocabulary at 4–5 years and vocabulary at 6–7 years. In alternative models estimated on 37–40 week births with individual gestational week dummy variables, we find few significant differences in outcomes between 37, 38 and 39 weeks relative to the reference case of 40 weeks for any of the outcomes.

Turning to estimates in Table 2, we observe significant negative relations between cesarean birth and measures of child cognitive development, up to a tenth of a standard deviation. The magnitude of the relations is similar across all measures, but only those with (NAPLAN) grammar, numeracy, reading, and writing at age 8–9, problem solving (MR) and vocabulary (PPVT) at age 4–5 are statistically significant at the 0.1 level or higher. To put the size of these relations into perspective, a tenth of a standard deviation is similar in magnitude to the estimated relation between gender and reading in NAPLAN at age 8–9 (reported in Table S5a) and effects estimated from improving teacher quality by one standard deviation and reducing average grade 3 class sizes by ten^50^.

Results from the first stage of our mediating analysis suggest that cesarean birth is significantly associated with lower rates of breastfeeding and higher rates of obesity and ADD (see Table S4 of online supporting material). Second stage results show that breastfeeding is significantly associated with higher cognitive performance, whereas ADD, ASD and obesity are significantly associated with lower levels of cognitive performance. Poor maternal general health is significantly associated with lower vocabulary at 4–5 years, but otherwise maternal health is not significantly linked to any of the cognitive measures.

Combining these results, breastfeeding, obesity and ADD are found to significantly mediate the relation between cesarean birth and child cognitive outcomes, although the effects size and significance vary (Table 3). Individually, the largest mediating effect is through reduced chances of breastfeeding, which explains 0.008 percentage points out of the 0.076 percentage point difference (or around 11%) of the gap in grade 3 NAPLAN writing scores. The total mediating effect, generated from regressions when all of the mediators are included together, account for between 25% for reading (p = 0.052) and 29% for numeracy (p = 0.021) of the estimated difference in cognitive outcomes by delivery mode. This still leaves at least 70% of the relations unexplained by these mediators.

**Table 3:** Mediating effects of breastfeeding and adverse child and maternal health outcomes on cognitive development.

Outcomes at 25	Breastfeeding^a^	Obesity^b^	ADD^c^	Asthma^c^	ASD^c^	Poor maternal physical health^d^	Poor maternal mental health^d^
*NAPLAN, 8–9*							
Numeracy	0.007**	0.006**	0.006*	0.000	0.005	0.001	0.000
	(0.003)	(0.003)	(0.003)	(0.000)	(0.003)	(0.001)	(0.000)
Grammar	0.006**	0.005**	0.007*	0.000	0.005	0.001	0.000
	(0.003)	(0.002)	(0.004)	(0.000)	(0.004)	(0.001)	(0.000)
Reading	0.008**	0.005**	0.006*	0.000	0.005	0.000	0.000
	(0.003)	(0.002)	(0.003)	(0.000)	(0.003)	(0.001)	(0.000)
Writing	0.003	0.002	0.008*	0.000	0.007	0.000	0.000
	(0.002)	(0.002)	(0.005)	(0.000)	(0.005)	(0.001)	(0.000)
*Survey-based cognitive measures*							
Vocabulary (PPVT), 4–5^e^	0.006**	0.001	0.002	0.000	0.003	0.002	0.000
	(0.003)	(0.002)	(0.002)	(0.001)	(0.002)	(0.002)	(0.001)
Problem solving (MR), 8–9	0.004*	0.005*	0.004*	0.000	0.002	0.001	0.000
	(0.002)	(0.003)	(0.002)	(0.000)	(0.002)	(0.002)	(0.000)

***p-value < 0.01; **p-value < 0.05; *p-value < 0.1. Standard errors are in parentheses. Results are estimated with adjustments for all confounders related to family socio-economic and perinatal risk factors. ^a^Breastfeeding is a binary measure of whether the mother report breastfeeding at 3 months after birth. ^b^Obesity is measured each year of the sample. A child is identified as obese using a binary measure of whether the child’s Body Mass Index is above the normal range at the time of cognitive testing (19.3 for those age 4–5 and 23 for 8–9). ^c^Asthma, attention deficit disorder (ADD) and autism spectrum disorder (ASD) are binary measures based on maternal reports of diagnosis. Diagnosis of ADD and ASD is only asked at age 8–9, whereas the diagnosis of asthma is asked each year. For asthma, we use information from the time of cognitive testing. ^d^Poor maternal health is self-reporting of fair or poor general health (4 or 5 on a 5-point scale of general health) and maternal mental health is ever experienced depressive symptoms in the last 4 weeks (a score below 3 on a Kessler 6-point scale of mental health). ^e^For the outcome of Vocabulary at age 4–5, there is no information on ADD and ASD at the time of testing because these conditions are typically not diagnosed until later. Information on diagnosis of ADD and ASD at 8–9 may still be a valid measure of the presence of these conditions at 4–5.

### Sensitivity test results

The estimated relations using the low-risk privately insured sample are generally larger than those estimated using the entire sample. A possible explanation is that by limiting the sample to privately-insured births we are reducing the unobserved confounding from the socio-economic advantage associated with cesarean-born children, which in the main analysis under-states the true effects of cesarean birth.

The Oster coefficients in Table 2 represent the possible lower-bound of any relation between cesarean birth and cognitive development measures under the conservative assumption that selection on unobserved covariates is the same as selection on observed covariates. An alternative interpretation is that the Oster parameter is the magnitude of the relation if adjustment from *unobserved* covariates is the same as adjustment from *observed* covariates in the model. With the exception of school readiness at 4–5 years and problem solving at 6–7 years, the Oster coefficients are much the same as the OLS regression coefficients estimated on the entire sample. Results of these sensitivity tests suggest that the estimated OLS relations are unlikely to be entirely driven by bias from unobserved confounding.

## Discussion

We find a negative relation between cesarean birth and a range of cognitive outcomes measured from ages 4 to 9. These relations are established after controlling for the socio-economic advantage associated with cesarean birth in Australia and a rich set of perinatal risk factors that (from meta-analyses)^9, 11, 27^ are commonly used in child health studies. Our results are consistent with results from the only previous study that we are aware of by Bentley *et al*.^31^, which found cesarean-born children had a 14% higher risk of being identified as being developmentally high risk at school starting age. While the magnitude of our estimated difference in outcomes is not large, up to a tenth of a standard deviation in national test scores in numeracy, they are large enough to warrant action. A tenth of a standard deviation in national test scores is comparable in size to differences related to gender, class size and teacher quality that are the focus of policy effort. Importantly, much of the estimated relations (around 70%) are found to be unrelated to lower rates of breastfeeding and adverse child and maternal health, including the diagnosis of child cognitive disorders.

We make two important contributions to the Bentley *et al*.^31^ study. First, our sensitivity analysis suggests that bias from unobserved confounding is unlikely to explain the results completely and that causal relations are plausible. This does not mean that we have established causal relations because bias from unobserved confounding is still possible. Potential perinatal risks not controlled for include inheritable genetic traits, such as a lack of maternal height, that may drive both cesarean birth (due to a relatively small pelvis (cephalopelvic disproportion))^51^ and child cognitive outcomes^52^. Second, our estimated relations persist long-term and are not confined to children with health problems. Bentley *et al*.^31^ used perinatal records linked to school starting-age measures of cognition and could not examine longer-term outcomes or quantify mediating effects. By showing that only a small part of the cognitive gap is explained by mediating factors, our results leave open the possibility that direct mechanisms, such as disturbed gut microbiota, may be important. However, we cannot rule-out the possibility that at least some of the residual effect is due to measurement error, for example, under-reporting of the presence of health conditions by the primary care giver, or that the mediating effects are biased by unobserved confounding.

Evidence presented in this study should motivate more research. Because experimentation is unsuitable, future studies may focus instead on instrumental variables estimation using large-scale linked hospital and child development administrative records. The instrumental variables approach exploits natural experiments, that is, random events that lead to variation in assignment to treatment without directly affecting outcomes. A limitation of this method is that the results only have a local treatment interpretation and cannot be generalized to those unaffected by the random event that led to assignment.

With the above caveats in mind, the key message to medical practitioners is to take a precautionary approach when formulating birth plans, especially when there are no apparent elevated health risks from vaginal birth. Informing mothers of the risks and benefits of cesarean birth should be a priority, which may be formalized by incorporating education sessions into practitioner guidelines.

## Electronic supplementary material

Supporting Information
