# Supplementary material for: The relation between cesarean birth and child cognitive development
Source: Sci Rep. 2017 Sep 13;7:11483. doi: 10.1038/s41598-017-10831-y (PMC5597642; doi:10.1038/s41598-017-10831-y)
Supplement: Supplementary file 1 — Supporting Information [file 41598_2017_10831_MOESM1_ESM.pdf]

The relation between cesarean birth and child cognitive development: Supporting material

Cain Polidano, Anna Zhu & Joel C. Bornstein

Table S1. OLS regression estimates of the relation between cesarean birth and NAPLAN scores, with and without the inclusion of imputed missing NAPLAN values

Table S2. OLS regression estimates of the relation between cesarean birth and cognitive measures, with and without the inclusion of extra perinatal risk factors

Table S3a. Full OLS regression results for NAPLAN measures, 8-9

Table S3b. Full OLS regression results for LSAC survey based measures

Table S4. Other mediation results

Table S1. OLS regression estimates of the relation between cesarean birth and NAPLAN scores, with and without the inclusion of imputed missing NAPLAN values

	With imputed NAPLAN values included[a]	With missing NAPLAN values excluded[b]
	(N=3,666)	(N=2,863)
Numeracy	-0.095***	-0.116***
	(0.034)	(0.040)
Grammar	-0.055*	-0.067*
	(0.033)	(0.039)
Spelling	-0.043	-0.051
	(0.033)	(0.040)
Reading	-0.076**	-0.096**
	(0.033)	(0.040)
Writing	-0.060*	-0.072*
	(0.031)	(0.038)

***Significant at 99%, **significant at 95%, *significant at 90%. Robust standard errors are in parentheses. Coefficients are measured in standard deviations. [a]These are the standard OLS regression results reported in the main text (Table 2) from the estimation of equation (1). It includes around 800 observations for which NAPLAN test scores were imputed. Imputations are made because these students have missing NAPLAN scores because they were no in grade 3 at the time of NAPLAN testing. We use multiple imputations to derive values based on information on prior performance in cognitive and non-cognitive tests available in the LSAC B cohort. [b]These results are generated from equation (1), but we omit observations with missing NAPLAN scores.

Table S2. OLS regression estimates of the relation between cesarean birth and cognitive measures, with and without the inclusion of extra perinatal risk factors

	**With standard perinatal controls**[a]	**With extra perinatal controls included**[b]
	(N=3,666)	(N=3,021)
Grade 3 NAPLAN		
Reading	-0.076**	-0.072*
	(0.033)	(0.037)
Writing	-0.060**	-0.077**
	(0.031)	(0.035)
Spelling	-0.043	-0.057
	(0.033)	(0.037)
Grammar	-0.055*	-0.058
	(0.033)	(0.037)
Numeracy	-0.095***	-0.086**
	(0.034)	(0.037)
Survey-based measures		
School preparedness (WAI), 4-5	-0.051	-0.066*
	(0.035)	(0.039)
Vocabulary (PPVT), 4-5	-0.087**	-0.105***
	(0.035)	(0.038)
Vocabulary (PPVT), 6-7	-0.050	-0.055
	(0.037)	(0.040)
Vocabulary (PPVT), 8-9	-0.047	-0.066
	(0.037)	(0.041)
Problem solving (MR), 6-7	-0.036	-0.021
	(0.117)	(0.129)
Problem solving (MR), 8-9	-0.069*	-0.057
	(0.039)	(0.044)

***Significant at 99%, **significant at 95%, *significant at 90%. Robust standard errors are in parentheses. Coefficients are measured in standard deviations. [a]These are the standard OLS regression results reported in the main text (Table 2) from the estimation of equation (1). In this treatment, we do not include some perinatal risk factors that are available in the data because they have large numbers of missing observations and are also observed after birth, which means that they may be an outcome of delivery mode. These risk factors are mother is a heavy daily drinker, mother is a regular smoker, maternal BMI within the normal range, fair to poor postnatal maternal health, ventilator used and intensive care used. [b]These are OLS regression estimates of equation (1), except that they include the extra perinatal controls listed above.

Table S3a. Full OLS regression results for NAPLAN measures, 8-9

	Reading	Writing	Spelling	Grammar	Numeracy
Cesarean birth	-0.076**	-0.060**	-0.043	-0.055*	-0.095***
	(.033)	(.031)	(.033)	(.033)	(.034)
**Controls**[a]					
Family characteristics					
Maternal age at birth	0.038	0.057**	0.025	0.065***	0.057**
	(.024)	(.024)	(.024)	(.025)	(.023)
Maternal age at birth^2	0.000	-0.001**	0.000	-0.001**	-0.001*
	(.000)	(.000)	(.000)	(.000)	(.000)
Number of older siblings (base: no older siblings)					
One	-0.172***	-0.067**	-0.141***	-0.133***	-0.097***
	(.035)	(.031)	(.034)	(.034)	(.035)
Two	-0.296***	-0.132***	-0.225***	-0.214***	-0.193***
	(.045)	(.041)	(.044)	(.044)	(.044)
Three or more	-0.346***	-0.224***	-0.389***	-0.337***	-0.312***
	(.063)	(.065)	(.067)	(.061)	(.065)
Female child	0.141***	0.414***	0.280***	0.254***	-0.148***
	(.029)	(.026)	(.028)	(.028)	(.029)
Either parent was born in a ’disadvantaged’ country[b]	-0.075	0.170***	0.264***	0.047	0.019
	(.056)	(.055)	(.062)	(.063)	(.064)
Mother is single	-0.270***	-0.308***	-0.296***	-0.255***	-0.148**
	(.066)	(.066)	(.067)	(.069)	(.065)
Mother is legally married	0.039	0.085**	0.047	0.081**	0.113***
	(.043)	(.038)	(.042)	(.041)	(.043)
Mother is employed	0.065	0.088**	0.041	0.084**	0.065
	(.043)	(.039)	(.041)	(.041)	(.042)
Hours of maternal employment per week	-0.002	-0.001	0.000	-0.001	-0.001
	(.001)	(.001)	(.001)	(.001)	(.001)
Mother’s highest qualification (base: VET qualification)[c]					
High school diploma or below[d]	-0.131***	-0.051	-0.100***	-0.052	-0.099**
	(.040)	(.036)	(.038)	(.039)	(.039)
Bachelor degree or higher	0.374***	0.281***	0.275***	0.396***	0.360***
	(.037)	(.034)	(.037)	(.035)	(.037)
State of residence (base: New South Wales)					
Victoria and Australian Capital Territory	0.017	-0.040	-0.181***	-0.036	-0.051
	(.038)	(.034)	(.037)	(.036)	(.038)
Western Australia	-0.114**	-0.230***	-0.258***	-0.203***	-0.168***
	(.052)	(.047)	(.049)	(.049)	(.052)
Queensland	-0.025	-0.156***	-0.229***	-0.085**	-0.159***

	(.041)	(.040)	(.041)	(.041)	(.042)
South Australia	-0.168***	-0.199***	-0.231***	-0.248***	-0.248***
	(.063)	(.050)	(.061)	(.065)	(.059)
Northern Territory	-0.173	-0.202	-0.384***	-0.180	-0.062
	(.117)	(.134)	(.117)	(.125)	(.134)
Tasmania	-0.125	-0.047	-0.303***	-0.205**	-0.167*
	(.101)	(.077)	(.090)	(.092)	(.098)
Outside metropolitan area	-0.093***	-0.162***	-0.146***	-0.116***	-0.122***
	(.031)	(.029)	(.030)	(.030)	(.031)
Private Health Insurance	0.131***	0.119***	0.091***	0.107***	0.130***
	(.034)	(.029)	(.032)	(.032)	(.034)
Perinatal factors					
Low birthweight (<2.5 kg)	-0.090	-0.105	-0.037	-0.101	-0.155*
	(.088)	(.074)	(.081)	(.076)	(.086)
IVF treatment used	0.019	-0.034	-0.055	-0.064	-0.073
	(.062)	(.060)	(.056)	(.056)	(.062)
Multiple infant pregnancy	-0.014	-0.006	0.161*	0.090	0.164*
	(0.091)	(0.080)	(0.096)	(0.089)	(0.091)
Head circumference of child (z-score)[e]	0.036*	0.038**	0.039*	0.035*	0.053***
	(.018)	(.017)	(.020)	(.018)	(.018)
Length of baby when born (z-score)[e]	0.017	0.014	0.027	0.011	0.042**
	(.017)	(.017)	(.017)	(.016)	(.018)
Blood pressure medication during pregnancy	-0.211**	-0.172*	-0.334***	-0.221**	-0.231**
	(.101)	(.091)	(.113)	(.098)	(.107)
Diabetes medication during pregnancy	-0.049	0.156	-0.130	0.031	0.064
	(.135)	(.149)	(.139)	(.113)	(.146)
Antibiotics medication during pregnancy	0.030	-0.013	0.006	-0.003	0.018
	(.046)	(.048)	(.048)	(.048)	(.046)
Weeks of gestation	0.003	-0.005	0.001	0.010	-0.009
	(.01)	(.009)	(.009)	(.009)	(.009)
Constant	-1.008*	-1.018**	-0.563	-1.736***	-0.735
	(.540)	(.496)	(.490)	(.550)	(.496)
Observations	3666	3666	3666	3666	3666
R^2	0.159	0.190	0.157	0.172	0.161

***Significant at 99%, **significant at 95%, *significant at 90%. Robust standard errors are in parentheses. Coefficients are measured in standard deviations. [a]All control variables are taken from wave 1 of the data (2004) when children are 0-1 year of age. [b]Disadvantaged countries are those identified by the Australian Bureau of Statistics Standard Australian Classification of Counties (2011), ABS cat. no. 1269.0. [c]Vocational education qualification is ISCED 1997 level 4B and 5A. [d]High school diploma includes vocational equivalent — International Standard Classification of Education (ISCED) 1997 level 3C. [e]Z-scores are based on Centre for Disease Control and Prevention (CDC) growth charts and are age and gender-adjusted.

Table S3b. Full OLS regression results for LSAC survey based measures

	School readiness 4-5	Vocab., 4-5	Vocab., 6-7	Vocab., 8-9	Problem solving, 6-7	Problem solving, 8-9
Cesarean birth	-0.051	-0.087**	-0.050	-0.047	-0.036	-0.069*
	(.035)	(.035)	(.037)	(.037)	(.117)	(.039)
**Controls**[a]						
Family characteristics						
Maternal age at birth	0.076***	0.076***	0.026	0.022	0.046	0.052*
	(.026)	(.028)	(.028)	(.029)	(.083)	(.030)
Maternal age at birth^2	-0.001***	-0.001**	0.000	0.000	0.000	-0.001
	(.000)	(.000)	(.001)	(.000)	(.001)	(.000)
Number of older siblings (base: no older siblings)						
One	-0.027	-0.088**	-0.204***	-0.169***	-0.303**	-0.098**
	(.036)	(.036)	(.038)	(.039)	(.121)	(.040)
Two	-0.226***	-0.150***	-0.416***	-0.374***	-0.391**	-0.265***
	(.045)	(.048)	(.050)	(.051)	(.161)	(.056)
Three or more	-0.338***	-0.282***	-0.410***	-0.423***	-0.862***	-0.310***
	(.068)	(.075)	(.077)	(.073)	(.224)	(.081)
Female child	0.648***	-0.142***	-0.088***	-0.106***	0.113	0.087**
	(.030)	(.030)	(.032)	(.033)	(.101)	(.034)
Either parent was born in a ’disadvantaged’ country[b]	0.107*	0.030	-0.283***	-0.315***	-0.325	0.018
	(.074)	(.078)	(.069)	(.069)	(.223)	(.074)
Mother is single	-0.144**	-0.170**	-0.102	-0.128	-0.310	-0.052
	(.072)	(.075)	(.080)	(.080)	(.227)	(.083)
Mother is legally married	0.113***	0.127***	-0.064	-0.062	0.143	0.088*
	(.043)	(.046)	(.048)	(.050)	(.149)	(.050)
Mother is employed	0.119***	0.076	0.118**	0.066	0.280*	0.070
	(.044)	(.046)	(.047)	(.048)	(.149)	(.05)
Hours of maternal employment per week	-0.002	-0.001	-0.002	-0.001	0.000	-0.002
	(.001)	(.001)	(.002)	(.002)	(.005)	(.002)
Mother’s highest qualification (base: VET qualification)[c]						
High school diploma or below[d]	-0.076*	-0.046	-0.128***	-0.083*	-0.319**	-0.062
	(.040)	(.044)	(.044)	(.046)	(.135)	(.048)
Bachelor degree or higher	0.171***	0.370***	0.263***	0.268***	0.648***	0.245***
	(.037)	(.039)	(.041)	(.042)	(.127)	(.044)
State of residence (base: New South Wales)						

Victoria and Australian Capital Territory	-0.122***	-0.038	0.038	0.085**	-0.278**	-0.063
	(.040)	(.041)	(.042)	(.043)	(.130)	(.045)
Western Australia	0.144***	-0.137**	0.118**	0.162***	-0.12	0.052
	(.053)	(.056)	(.060)	(.062)	(.191)	(.061)
Queensland	-0.133***	-0.157***	-0.018	0.051	-0.048	0.032
	(.043)	(.043)	(.044)	(.045)	(.144)	(.049)
South Australia	0.060	-0.269***	0.382***	0.308***	0.313	-0.090
	(.060)	(.061)	(.070)	(.077)	(.218)	(.072)
Northern Territory	0.098	0.006	0.213	0.079	0.347	0.017
	(.113)	(.127)	(.118)	(.136)	(.338)	(.140)
Tasmania	0.110	0.413***	0.349***	0.346***	0.368	0.045
	(.097)	(.107)	(.113)	(.106)	(.367)	(.100)
Outside metropolitan area	-0.161***	-0.040	-0.023	-0.03	-0.374***	-0.108***
	(.032)	(.033)	(.034)	(.035)	(.106)	(.037)
Private Health Insurance	0.089***	0.253***	0.142***	0.190***	0.091	0.049
	(.034)	(.036)	(.037)	(.038)	(.114)	(.040)
Perinatal factors						
Low birthweight (<2.5 kg)	-0.249***	-0.206**	-0.159*	-0.050	0.008	-0.006
	(.085)	(.094)	(.090)	(.104)	(.276)	(.100)
IVF treatment used	-0.089	-0.081	-0.053	-0.096	-0.318	-0.021
	(.062)	(.064)	(.064)	(.077)	(.211)	(.075)
Multiple infant pregnancy	0.149	-0.021	-0.007	0.099	0.222	0.078
	(.084)	(.087)	(.103)	(.103)	(.343)	(.095)
Head circumference of child (Z-score)[e]	0.031	0.032	0.042**	0.000	0.066	0.047**
	(.019)	(.020)	(.022)	(.021)	(.065)	(.023)
Length of baby when born (z-score) [e]	0.031*	0.015	-0.027	0.036*	0.143**	0.063***
	(.018)	(.019)	(.020)	(.019)	(.060)	(.022)
Blood pressure medication during pregnancy	-0.345***	-0.089	-0.071	0.008	-0.024	-0.064
	(.105)	(.110)	(.115)	(.101)	(.372)	(.117)
Diabetes medication during pregnancy	0.054	-0.245	0.131	0.008	-0.009	0.041
	(.176)	(.178)	(.136)	(.151)	(.531)	(.147)
Antibiotics medication during pregnancy	-0.044	0.079	0.038	-0.004	-0.008	-0.044
	(.051)	(.049)	(.053)	(.052)	(.162)	(.054)
Weeks of gestation	0.020*	-0.013	-0.002	-0.014	-0.001	-0.013
	(.010)	(.011)	(.011)	(.012)	(.034)	(.011)
Constant	-2.286***	-0.976	-0.597	-0.064	9.446***	-0.542
	(.544)	(.608)	(.626)	(.634)	(1.803)	(.629)
Observations	3666	3635	3428	3287	3428	3275

R^2	0.197	0.145	0.122	0.110	0.061	0.069

***Significant at 99%, **significant at 95%, *significant at 90%. Robust standard errors are in parentheses. Coefficients are measured in standard deviations. [a]All control variables are taken from wave 1 of the data (2004) when children are 0-1 year of age. [b]Disadvantaged countries are those identified by the Australian Bureau of Statistics Standard Australian Classification of Counties (2011), ABS cat. no. 1269.0. [c]Vocational education qualification is ISCED 1997 level 4B and 5A. [d]High school diploma includes vocational equivalent — International Standard Classification of Education (ISCED) 1997 level 3C. [e]Z-scores are based on Centre for Disease Control and Prevention (CDC) growth charts and are age and gender-adjusted.

Table S4. Adjusted relations between cesarean birth and mediating variables and mediating variables and outcomes

Mediating variables	Independent variable	Outcome variables					
		NAPLAN 8-9		Survey-administered measures			
	Cesarean birth	Numeracy	Grammar	Reading	Writing	Vocabulary (PPVT), 4-5[e]	Problem solving (MR), 8-9
Breastfeeding[a]	-0.047***	0.155***	0.136***	0.160***	0.061*	0.116***	0.092**
	(0.017)	(0.037)	(0.036)	(0.037)	(0.034)	(0.038)	(0.040)
Obesity[b]	0.051***	-0.122***	-0.096***	-0.099***	-0.040	0.016	-0.108***
	(0.017)	(0.036)	(0.037)	(0.038)	(0.033)	(0.040)	(0.041)
Asthma[c]	0.005	-0.022	0.027	-0.014	-0.003	-0.067	0.007
	(0.014)	(0.044)	(0.043)	(0.043)	(0.040)	(0.049)	(0.049)
ADD[c]	0.012*	-0.515***	-0.577***	-0.513***	-0.678***	-0.247**	-0.349***
	(0.006)	(0.092)	(0.098)	(0.101)	(0.115)	(0.099)	(0.119)
ASD[c]	0.012	-0.445***	-0.454***	-0.419***	-0.607***	-0.323***	-0.226*
	(0.007)	(0.095)	(0.100)	(0.105)	(0.107)	(0.107)	(0.121)
Poor maternal general health[d]	0.013	0.048	0.041	0.029	-0.006	-0.160**	0.111
	(0.011)	(0.068)	(0.065)	(0.068)	(0.068)	(0.067)	(0.072)
Poor maternal mental health[d]	-0.001	-0.068	0.034	-0.023	0.030	-0.166	-0.016
	(0.005)	(0.125)	(0.112)	(0.124)	(0.099)	(0.126)	(0.148)

***p-value < 0.01; **p-value < 0.05; *p-value < 0.1. Robust standard errors are in parentheses. Results are estimated with adjustments for all confounders related to family socio-economic and perinatal risk factors. [a]Breastfeeding is a binary measure of whether the mother report breastfeeding at 3 months after birth. [b]Obesity is a binary measure of whether the child's Body Mass Index is above the normal range (19.3 for those age 4-5 and 23 for 8-9). [c]Asthma, attention deficit disorder (ADD) and autism spectrum disorder (ASD) are binary measures based on maternal reports of diagnosis at age 8-9, except for asthma, which is at age 4-5. [d]Poor maternal health is self-reporting of fair or poor general health (4 or 5 on a 5-point scale of general health) and maternal mental health is ever experienced depressive symptoms in the last 4 weeks (a score below 3 on a Kessler 6-point scale of mental health). [e]For the outcome of Vocabulary at age 4-5, there is no information on ADD and ASD at the time of testing because these conditions are typically not diagnosed until later. Information on diagnosis of ADD and ASD at 8-9 may still be a valid measure of the presence of these conditions at 4-5.
